# A severity-anchored, patient-reported outcome framework for clinical trials in post-stroke dysphagia

**DOI:** 10.3389/fneur.2026.1826727

**Published:** 2026-08-24

**Authors:** Adnan I. Qureshi, Gunjanpreet Kaur, Lourdes Carhuapoma, Pashmeen Lakhani, Maureen A. Lefton-Greif, David R. Mehr, Lisa A. Royse, Nae-Yuh Wang, Kamran Zahoor, Daniel F. Hanley

**Affiliations:** 1 Zeenat Qureshi Stroke Institute, Columbia, MO, United States; 2 Department of Neurology, University of Missouri, Columbia, MO, United States; 3 BIOS Clinical Trials Coordinating Center, Trial Innovation Center, Johns Hopkins University School of Medicine, Baltimore, MD, United States; 4 Department of Pediatrics, Eudowood Division of Pediatric Respiratory Sciences, Johns Hopkins University School of Medicine, Baltimore, MD, United States; 5 Department of Otolaryngology-Head and Neck Surgery, The Johns Hopkins University School of Medicine, Baltimore, MD, United States; 6 Department of Physical Medicine and Rehabilitation, The Johns Hopkins University School of Medicine, Baltimore, MD, United States; 7 Department of Family and Community Medicine, University of Missouri, Columbia, MO, United States; 8 Department of Orthopedic Surgery, University of Missouri, Columbia, MO, United States; 9 Johns Hopkins University School of Medicine, Baltimore, MD, United States

**Keywords:** clinical trials, patient-centered outcomes, post-stroke dysphagia, stepped-wedge design, stroke

## Abstract

**Background:**

Post-stroke dysphagia is common and is associated with aspiration pneumonia, malnutrition, longer hospitalization, and reduced quality of life. Trials evaluating interventions for post-stroke dysphagia face persistent challenges in selecting endpoints that are both patient-centered and methodologically robust.

**Objectives:**

To derive and justify a patient-reported outcome (PRO) endpoint for stroke-related dysphagia that supports clinically meaningful interpretation, statistical efficiency, and alignment with regulatory expectations.

**Methods:**

We synthesized regulatory guidance, outcomes-research standards, and stroke-specific evidence to develop a dichotomous, status-based PRO responder endpoint at 90 days post-stroke that integrates swallowing-specific quality of life (SWAL-QOL) and global health-related quality of life (EuroQol-5D; EQ-5D). Responder definitions were calibrated to baseline dysphagia severity using swallowing-relevant National Institutes of Health Stroke Scale (NIHSS) subcomponents assessed in the early post-acute period. Monte Carlo simulations (10,000 simulated patients with post-stroke dysphagia) compared performance of the combined responder endpoint versus (i) continuous SWAL-QOL and (ii) utility-weighted modified Rankin Scale (UW-mRS) analyses under clinically realistic severity distributions and treatment effects. Sample size requirements were estimated for individually randomized and stepped-wedge designs.

**Results:**

Severity-anchored responder endpoints were more statistically efficient and more interpretable than continuous or change-based analyses in heterogeneous acute stroke populations. In simulation, combined SWAL-QOL + EQ-5D responder rates increased from 42.7% (control) to 66.2% (treatment), an absolute difference of 23.5% (odds ratio 2.58, 95% CI 2.38–2.81; number needed to treat ≈ 4–5), with the largest effects in moderate and severe dysphagia. In contrast, mean SWAL-QOL improved by 8.3 points (near or below commonly cited minimal clinically important difference thresholds), and mean UW-mRS improved by 0.017, indicating limited sensitivity of global disability measures.

**Conclusion:**

A severity-calibrated, dichotomous PRO responder endpoint combining SWAL-QOL and EQ-5D provides a patient-centered, statistically efficient, and methodologically robust framework for evaluating therapeutic interventions in post-stroke dysphagia.

## Introduction

Post-stroke dysphagia is common and associated with aspiration pneumonia, malnutrition, prolonged hospitalization, and reduced quality of life (1, 2). In the United States, an estimated 352,742 patients with ischemic stroke experience dysphagia each year (1). In published economic models, 1-year acute hospital and post-hospitalization costs were estimated at $67,100 to $112,400 per patient with dysphagia versus $51,979.8 to $54,031.0 per patient without dysphagia (1). Clinical practice guidelines emphasize early identification and management of dysphagia, including screening, nutritional support, and risk mitigation (2–5). For patients with persistent dysphagia (approximately 15–20% of patients with stroke) (2, 3, 6–9), guideline-recommended options may include enteral feeding via percutaneous endoscopic gastrostomy (PEG) to support hydration and nutrition.

Standard care emphasizes early screening, nutritional support, behavioral swallowing therapy, and preventive measures such as oral hygiene, but these approaches do not directly accelerate neurologic recovery of swallowing function. Evidence supporting many existing interventions is heterogeneous, and no dysphagia-modifying treatment has demonstrated consistent efficacy. Adjunctive neurostimulation techniques (e.g., pharyngeal electrical stimulation, non-invasive brain stimulation) show promise in selected populations (10, 11), but results are variable, patient selection remains uncertain, and integration into routine care is limited. Consequently, a substantial proportion of patients experience persistent dysphagia requiring long-term enteral feeding, with significant patient and caregiver burden (12). These gaps underscore the need for novel therapeutic strategies and for clinical trials designed to evaluate meaningful patient benefits.

Developing and testing new interventions within methodologically robust, patient-centered trial frameworks is essential to move beyond supportive care and improve long-term outcomes. While physiologic swallowing assessments and clinician-reported scales provide important information about swallowing mechanics and safety, they do not fully capture the lived experience of patients with dysphagia, including eating burden, social participation, emotional distress, and perceived recovery (13, 14). Patient-reported outcomes (PROs) are therefore increasingly recognized as essential endpoints in dysphagia research and stroke rehabilitation trials (14, 15).

However, deriving appropriate PRO endpoints for acute stroke dysphagia presents unique challenges. In the early post-stroke period, neurologic instability, aphasia, reduced consciousness, and medical complications frequently limit reliable baseline PRO assessment (16). In addition, recovery trajectories are heterogeneous, with ceiling and floor effects that complicate interpretation of mean or median score changes (17). These challenges are amplified in trials evaluating timing-dependent interventions (e.g., early versus delayed gastrostomy tube placement), where baseline PRO measurement may be differentially feasible or temporally confounded by treatment exposure (18).

**Figure 1:**
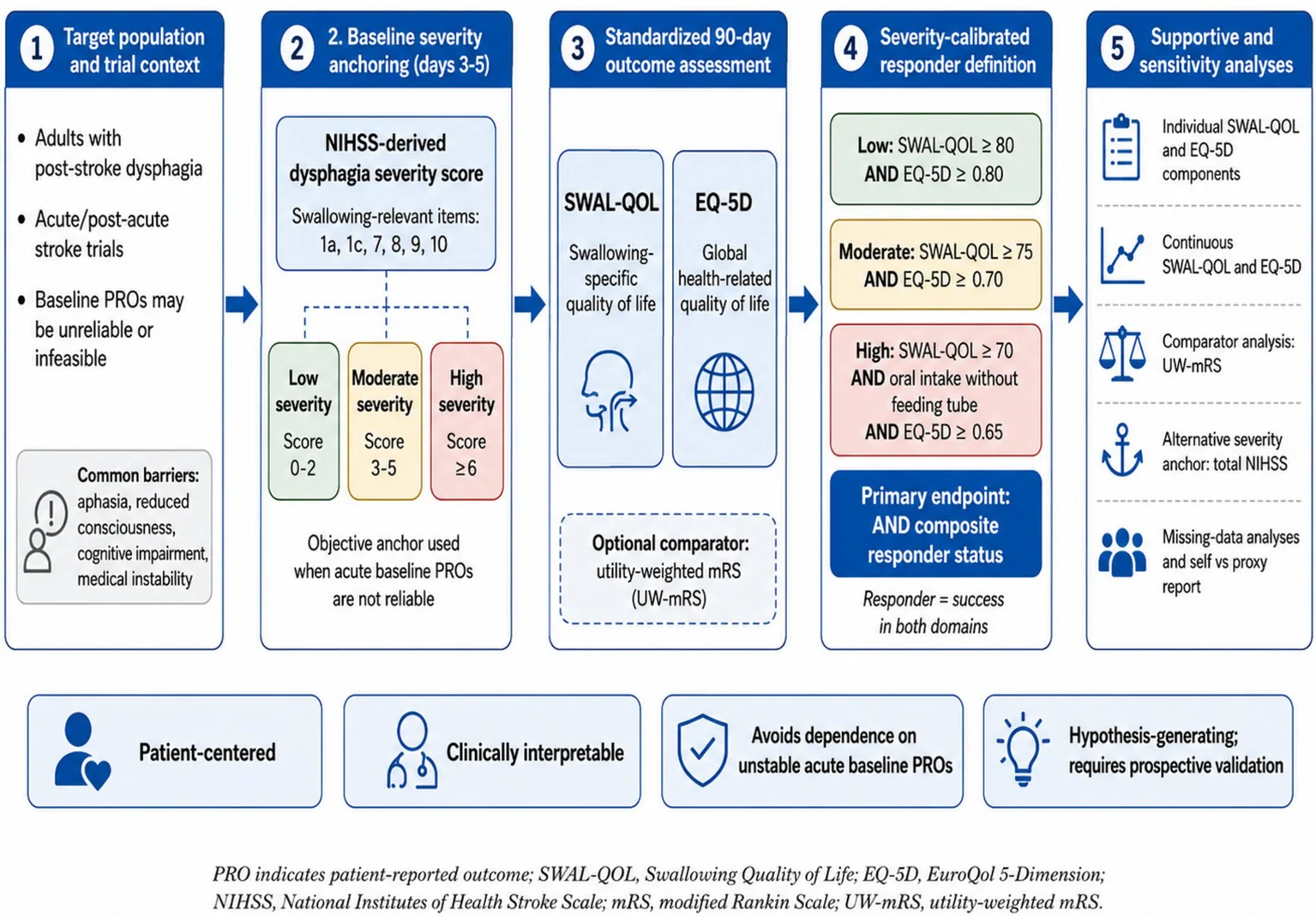
Severity-anchored PRO framework for post-stroke dysphagia trials. Baseline dysphagia severity is anchored using an NIHSS-derived score, and 90-day outcomes are assessed with SWAL-QOL and EQ-5D. Responder status is defined by severity-calibrated thresholds requiring success in both domains, with supportive analyses including continuous outcomes, UW-mRS, alternative severity anchors, and missing-data analyses.

We therefore propose a severity-anchored, status-based PRO responder framework for post-stroke dysphagia trials that is patient-centered, clinically interpretable, and statistically efficient, and that aligns with contemporary regulatory and outcomes-research expectations. A summary Figure 1 has been added to graphically synthesize the overall framework, including baseline severity stratification, 90-day outcome generation, severity-calibrated responder classification, and comparator analyses.

## Methods

### Conceptual and regulatory framework

We synthesized regulatory guidance, outcomes-research standards, and stroke-specific evidence to derive a PRO endpoint strategy suitable for acute stroke dysphagia trials. Guidance from the U.S. Food and Drug Administration (FDA) (19), the Patient-Centered Outcomes Research Institute (PCORI) (20), the European Medicines Agency (EMA) (21), and the International Society for Pharmacoeconomics and Outcomes Research (ISPOR) (22) emphasizes that PROs should measure concepts meaningful to patients, use validated instruments, and be assessed at clinically interpretable time points. FDA and EMA guidance cautions against relying on acute-phase baseline PRO measurements in neurologic conditions when patients may be unstable or unable to self-report, because such baselines can introduce bias and inflate apparent change (19, 21, 23). PCORI methodology standards emphasize patient-centeredness, feasibility, and equity (24), and support status-based or responder endpoints when baseline PRO collection is impractical, provided that thresholds are prespecified, clinically justified, and supported by sensitivity analyses (20, 24).

Across these guidance sources, common principles support PRO strategies that (1) prioritize meaningful patient benefit at standardized recovery milestones (e.g., 90 days), (2) avoid dependence on unreliable acute baselines, (3) account for baseline severity using objective clinical anchors, and (4) prespecify clinically meaningful responder criteria and missing-data strategies. In neurologic and rehabilitation contexts, this framework favors status-based or calibrated responder endpoints, supplemented by adjusted analyses and secondary continuous outcomes.

### Selection of patient-reported outcomes

PRO measures were selected in accordance with the National Institute of Neurological Disorders and Stroke (NINDS) Common Data Elements (CDE) framework (25), which recommends measures with established reliability, responsiveness, and clinical relevance. Use of CDE-based outcomes facilitates cross-study comparison and aligns endpoint selection with federal research standards and contemporary regulatory guidance (25, 26). Within the NINDS CDE framework, dysphagia-related measures include validated swallowing-specific instruments and generic health-related quality of life measures relevant to stroke recovery.

### Swallowing-specific quality of life (SWAL-QOL)

The Swallowing Quality of Life (SWAL-QOL) questionnaire was selected as the disease-specific PRO (27). SWAL-QOL score ranges from 0 to 100, with higher scores indicating better quality of life and captures patient-centered domains not reflected by physiologic or clinician-reported assessments, including eating burden, social participation, and emotional impact (28). Because physiologic swallowing improvement does not consistently translate into meaningful functional recovery, SWAL-QOL provides essential insight into whether dysphagia affects daily life and perceived recovery (29).

### Global health-related quality of life (EQ-5D)

The EuroQol 5-Dimension instrument (EQ-5D) was selected to assess global health-related quality of life (30, 31). EQ-5D evaluates domains central to stroke recovery, including mobility, self-care, usual activities, pain/discomfort, and anxiety/depression (32). EQ-5D index values are anchored at 1 = full health and 0 = dead. While SWAL-QOL captures swallowing-specific impact, EQ-5D provides a complementary measure of overall health status and is widely accepted for regulatory and health-technology assessment purposes (33).

### Primary endpoint definition

Swallowing-specific and global quality of life outcomes were operationalized as a dichotomous, status-based responder endpoint assessed at 90 days post-stroke, integrating SWAL-QOL and EQ-5D. Dichotomization of outcomes is an established analytic strategy in stroke research, analogous to dichotomized modified Rankin Scale (mRS) outcomes, and can facilitate interpretability across heterogeneous severity strata (34, 35). A status-based endpoint at a standardized recovery milestone avoids reliance on unstable or infeasible acute baseline PRO measurement, which can introduce bias in change-from-baseline analyses (19, 23, 36–39).

Mean or median PRO comparisons assume linearity and comparable interpretability of score differences across patients (17, 40). These assumptions are rarely met in acute stroke dysphagia, where baseline severity is heterogeneous and recovery trajectories vary widely (12, 41). Continuous PRO measures may exhibit ceiling effects in mildly affected patients and floor effects in severely affected patients, obscuring clinically meaningful recovery patterns (17, 42, 43). A responder endpoint mitigates these limitations by focusing on the proportion of patients who cross prespecified, clinically meaningful thresholds rather than average score changes.

### Composite responder definition

The primary endpoint was defined as an “AND” composite: participants are classified as responders only if they achieve both a good SWAL-QOL outcome and a good EQ-5D outcome at 90 days. This concordant definition aims to ensure that swallowing-specific recovery is accompanied by meaningful global health benefit and to reduce false-positive classification that may occur when success is defined in either domain alone (22, 33, 44). Analyses of individual PRO components and continuous outcomes remain important secondary and supportive endpoints for mechanistic interpretation.

In multicenter trials, the framework would require standardized assessment windows, uniform administration guidance, assessor training, and centralized automated scoring. When patients cannot reliably self-report because of aphasia, cognitive impairment, or severe stroke, validated proxy assessment may be used where appropriate, with the respondent type documented. Because inability to complete PRO measures may be associated with greater stroke severity and poorer outcomes, missingness will be considered potentially informative. The primary analysis may conservatively classify missing outcomes as non-response, supplemented by multiple imputation, pattern-mixture analyses, and sensitivity analyses stratified by self- versus proxy-report.

### Baseline severity anchoring with National Institutes of Health Stroke Scale subcomponents

Responder thresholds were calibrated to baseline dysphagia severity using swallowing-relevant National Institutes of Health Stroke Scale (NIHSS) subcomponents assessed in the early post-acute period (days 3–5 post-stroke). NIHSS is a validated and widely used measure of stroke severity and provides an objective clinical anchor when baseline PRO assessment is unreliable or infeasible in the acute setting (45, 46). Specific NIHSS domains are mechanistically linked to swallowing safety and recovery, including level of consciousness and command following, language impairment and dysarthria, sensory deficits associated with silent aspiration, and limb ataxia reflecting cerebellar or brainstem involvement (47, 48). Prior studies support NIHSS as a clinically meaningful predictor of dysphagia presence, severity, and recovery after stroke (42, 49–55).

A prespecified dysphagia-relevant severity construct was derived from NIHSS score items associated with swallowing dysfunction: level of consciousness (Item 1a), ability to follow commands (Item 1c), limb ataxia (Item 7), sensory loss (Item 8), language impairment (Item 9), and dysarthria (Item 10) (56). Each item was dichotomized (score 0 vs. ≥ 1) and combined using prespecified weights to generate a composite dysphagia severity score, which was then categorized into low-, moderate-, and high-risk strata.

The severity score is intended to function as a baseline severity anchor rather than a diagnostic test. Based on published cohort studies and prediction models, expected discriminative performance for clinically significant dysphagia is approximately Area under the curve (AUC) 0.75–0.85 using clinical reference standards and AUC 0.80–0.90 when instrumental severity measures are used (52, 53). Sensitivity analyses comparing the dysphagia severity score with total NIHSS score may be used to confirm robustness (see Tables 1, 2).

**Table 1:** Evidence supporting NIHSS-based dysphagia risk and severity assessment after stroke.

Study (year)	Population/*N*	Dysphagia outcome	NIHSS predictors evaluated	Key effect sizes/performance	Key conclusion
Okubo et al. (2012) (49)	Acute ischemic stroke; *N* ≈ 200	Clinical dysphagia (bedside)	NIHSS total score	NIHSS ≥12: Sensitivity ~88%, Specificity ~85%	NIHSS total score is a strong early predictor of dysphagia risk.
Labeit et al. (2018) (52)	Acute stroke; *N* ≈ 300	Objective dysphagia severity (FEDSS)	NIHSS total; infratentorial vs. supratentorial	NIHSS correlated with FEDSS (R^2^ ≈ 0.7–0.8); lower cutoffs for infratentorial strokes	NIHSS correlates with severity of dysphagia; thresholds vary by stroke location.
Jeyaseelan et al. (2015) (53)	Inpatient rehabilitation; *N* ≈ 150	Clinically relevant dysphagia	NIHSS total score	NIHSS >9 independently predicted dysphagia	NIHSS useful for identifying persistent/clinically significant dysphagia.
Lin et al. (2019) (54)	Acute stroke with dysphagia; *N* ≈ 120	Early dysphagia recovery	NIHSS subitems: Facial palsy (4), Language (9)	Subitems independently associated with recovery (ORs ~ 1.5–2.0)	Specific NIHSS subcomponents predict recovery beyond total NIHSS.
Kim et al. (2020) (Korean nationwide) (42)	Ischemic stroke; *N* > 10,000	Dysphagia diagnosis	NIHSS total; age; location	NIHSS independently associated with dysphagia (adjusted OR per point ~1.2–1.3)	NIHSS is a robust, population-level predictor of dysphagia.
Ye et al. (2024) ML prediction models (2020–2024) (55)	Multicenter cohorts	Early dysphagia	NIHSS total + subcomponents	NIHSS among top predictors; AUCs ~0.80–0.90	NIHSS consistently ranks as a key predictor in multivariable models.

FEDSS = Fiberoptic Endoscopic Dysphagia Severity Score; NIHSS = National Institutes of Health Stroke Scale; OR = odds ratio; AUC = area under the curve.

**Table 2:** Dysphagia severity score based on NIH Stroke Scale (NIHSS) subcomponents.

NIHSS item	Neurologic domain	Scoring rule	Points assigned	Rationale for dysphagia
1a—Level of consciousness	Alertness/arousal	0 = normal; ≥1 = impaired	2	Reduced consciousness compromises airway protection and swallow initiation
1c—LOC commands	Attention/executive function	0 = follows commands; ≥1 = impaired	1	Inability to follow commands limits safe swallow assessment and compensatory strategies
9—Best language	Cortical language function	0 = no aphasia; ≥1 = aphasia	2	Strong predictor of dysphagia severity and persistence; reflects dominant cortical involvement
10—Dysarthria	Bulbar motor control	0 = normal; ≥1 = impaired	1	Indicates corticobulbar or brainstem dysfunction affecting oropharyngeal control
7—Limb ataxia	Cerebellar/brainstem coordination	0 = absent; ≥1 = present	1	Associated with impaired swallow timing and coordination, especially posterior circulation
8—Sensory	Sensory feedback	0 = normal; ≥1 = impaired	1	Reduced pharyngeal sensation increases silent aspiration risk

Severity stratification for NIHSS-derived dysphagia score		
Total score	Dysphagia severity category	Typical clinical phenotype
0–2	Low risk	Mild dysphagia; oral intake usually preserved
3–5	Moderate risk	High aspiration risk; variable oral intake
≥6	High risk	Severe dysphagia; often NPO or feeding tube-dependent

Maximum score: 8 points. NPO=Nothing by mouth (from the Latin nil per os).

### Severity-calibrated status thresholds

Good outcomes were defined using severity-calibrated status thresholds for SWAL-QOL and EQ-5D anchored to NIHSS-derived dysphagia severity (Table 3). These thresholds have not been prospectively validated and should not be interpreted as established minimal clinically important differences or definitive responder criteria. Rather, they were developed as hypothesis-generating values for evaluating the feasibility and statistical operating characteristics of a severity-calibrated responder framework. Patients with low severity were required to achieve near-normal status, whereas those with moderate or high severity were evaluated using lower absolute thresholds that reflect realistic recovery trajectories and mitigate ceiling and floor effects (17, 41). For the highest-risk group, classification of a good swallowing outcome also required oral intake without a feeding tube to ensure that responder status reflects functional recovery rather than adaptation to enteral feeding.

**Table 3:** Severity-calibrated status thresholds for SWAL-QOL and EQ-5D anchored to NIHSS-derived dysphagia severity.

(A) SWAL-QOL: Severity-calibrated status thresholds		
Dysphagia severity	Definition of “Good SWAL-QOL outcome” at 90 Days	Clinical rationale
SWAL-QOL responder thresholds		
Low risk (0–2)	SWAL-QOL ≥ 80	Near-normal swallowing-related quality of life expected
Moderate risk (3–5)	SWAL-QOL ≥ 75	Meaningful swallowing recovery short of full normalization
High risk (≥6)	SWAL-QOL ≥ 70 AND oral intake without feeding tube	Reflects substantial recovery in severe dysphagia
Key points: Absolute thresholds reflect achievable recovery by severity. Feeding tube removal is incorporated only in the high-risk group, where it is clinically decisive. No baseline PRO is required.		

(B) EQ-5D-5L: Severity-calibrated status thresholds		
Dysphagia severity	Definition of “Good EQ-5D outcome” at 90 Days	Clinical rationale
EQ-5D responder thresholds		
Low risk (0–2)	EQ-5D index ≥ 0.80	Near-independent global health
Moderate risk (3–5)	EQ-5D index ≥ 0.70	Functional independence with residual limitations
High risk (≥6)	EQ-5D index ≥ 0.65	Meaningful global recovery in severe stroke
Key points: Thresholds correspond to clinically interpretable health states. EQ-5D is not tied to baseline values, avoiding acute-phase bias. The proposed values are prespecified, hypothesis-generating candidate thresholds. They have not been prospectively validated as minimal clinically important differences or definitive responder cutoffs and should be evaluated using alternative cutoffs and continuous outcome analyses.		

### Statistical analysis and sample size planning

Sample size planning assumed a conservative control responder rate of 40% (proportion achieving a good composite responder outcome at 90 days) and a prespecified clinically important target absolute difference of 15 percentage points. The 15-point target was selected to represent an effect large enough to justify adoption given intervention burden and expected usual-care recovery, while remaining plausible based on available evidence. This magnitude corresponds to NNT ≈ 7 (1/0.15), i.e., ~1 additional responder for every ~7 participants treated.

Individually randomized trial (1:1). The primary analysis will compare 90-day responder status using logistic regression under the intention-to-treat principle, with adjustment for baseline severity strata as appropriate. With two-sided *α* = 0.05 and 80% power, approximately 350 evaluable participants are required (≈175/group); inflating for 10–15% attrition yields an enrollment target of ~400–415.

Cluster-based delivery. For interventions delivered at the stroke unit/health-system level, parallel cluster and stepped-wedge cluster designs were evaluated using generalized linear mixed-effects models, accounting for clustering (and secular time effects for stepped-wedge designs) (57). Planning assumed ICC = 0.03 (58–60). For planning, variance inflation due to clustering was summarized as:

Design effect (DE) ≈ 1 + (m − 1) × ICC, where *m* is the average cluster size (or cluster-period size, as applicable).

- Parallel cluster randomized trial: If m ≈ 20/site, DE ≈ 1.57, implying ~550 evaluable participants (≈28 sites). If the number of sites is fixed at 20 (10/arm), targeting ~37/site (total ~740 evaluable; DE ≈ 2.08) provides modest margin; with 10–15% attrition, enrollment increases to ~820–870 (≈41–44/site).
- Stepped-wedge cluster randomized trial: The planned stepped-wedge design includes 20 sites, 5 sequences (4 sites/sequence), and 6 periods. Under cross-sectional recruitment, the target is 8 participants/site-period (rounded from ~7.x), yielding *N* = 960 total (20 × 6 × 8; ~48/site). The primary model will include a random effect for site and fixed effects for period to adjust for secular time trends (57).

Secondary outcomes and sensitivity analyses. Continuous SWAL-QOL and EQ-5D at 90 days will be analyzed as secondary endpoints and interpreted using published MCID estimates (17, 33, 41, 61). Prespecified sensitivity analyses will assess robustness to responder thresholds, alternative severity anchors (e.g., total NIHSS score), and missing-data assumptions (e.g., missing = non-responder; multiple imputation under missing at random (MAR)), with exploratory per-protocol and change-from-baseline analyses where baseline PROs are reliable (62, 63) (Tables 4, 5).

**Table 4:** Comparison of sample size requirements: dichotomous responder versus continuous mean comparison.

Endpoint type	Outcome definition	Key assumptions	Clinically meaningful effect	Total sample size for 80% power	Key limitations
Dichotomous responder (preferred)	NIHSS-anchored combined SWAL-QOL + EQ-5D responder at 90 days	Control responder rate = 40% Intervention responder rate = 55% α = 0.05	15% absolute increase in meaningful recovery (OR ≈ 1.8; NNT ≈ 7)	420–450	Conservative but highly interpretable
Continuous mean comparison (optimistic variance)	Mean SWAL-QOL difference at 90 days	Δ = 8 points SD = 20 *α* = 0.05	≈ MCID	~100	Unrealistically low variance
Continuous mean comparison (realistic variance)	Mean SWAL-QOL difference at 90 days	Δ = 8 points SD = 25–30 α = 0.05	≈ MCID	150–220	Sensitive to heterogeneity and ceiling/floor effects
Continuous mean comparison (change from baseline)	Mean change in SWAL-QOL	*Δ* = 10 points SD = 30 + Missing baseline PROs	≈ MCID	>300–400	Biased; unstable baselines
Median comparison	Median SWAL-QOL at 90 days	Nonparametric test	Undefined	Often >400	Low power; ignores tail recovery
Stepped-wedge dichotomous responder	NIHSS-anchored responder	ICC = 0.03 6 steps	15% absolute increase	660–720	Inflation due to clustering
Stepped-wedge continuous mean	Mean SWAL-QOL	SD = 25–30 ICC = 0.03	8–10 points	>900	Poor efficiency

Continuous endpoint sample sizes assume stable baselines and variance as specified; under realistic acute stroke conditions, variance inflation and missing baseline PROs may reduce efficiency of mean-based analyses. SWAL-QOL=Quality of Life in Swallowing Disorders questionnaire, EQ-5D=EuroQol 5-Dimension, SD=Standard Deviation, ICC=Intraclass Correlation Coefficient, PRO=Patient-reported outcomes, OR= Odds ratio, NNT=number needed to treat, MCID=Minimal Clinically Important Difference.

**Table 5:** Sensitivity analyses: purpose and methodologic rationale.

Sensitivity analysis	Primary question addressed	Methodologic concern tested	Interpretation if consistent with primary analysis
Uniform responder thresholds (no severity calibration)	Does the treatment effect depend on NIHSS-based calibration?	Dependence on severity-specific cutpoints	Confirms that benefit is not an artifact of calibrated thresholds
Continuous SWAL-QOL at 90 days	Is benefit visible on the raw disease-specific scale?	Loss of information from dichotomization	Supports that responder effects reflect underlying score shifts
Continuous EQ-5D at 90 days	Does global health improve on the continuous scale?	Over-reliance on binary health-state definitions	Demonstrates concordant improvement in overall quality of life
Total NIHSS instead of dysphagia severity score	Is the result sensitive to the choice of NIHSS anchor?	Model dependence on selected subcomponents	Shows robustness to alternative severity adjustment
Feeding tube status alone (descriptive)	Is tube removal driving the primary result?	Confounding by nutrition management	Clarifies mechanism without overstating causality
Change-from-baseline analyses (subset only)	Are results similar when baseline PROs are available?	Baseline instability and missingness bias	Supports consistency while acknowledging limitations of change scores
Missing = non-responder	Are results sensitive to conservative missing-data assumptions?	Bias from incomplete follow-up	Confirms findings are not driven by optimistic imputation
Multiple imputation for missing outcomes	Are results robust under MAR assumptions?	Missing data mechanism uncertainty	Demonstrates stability across plausible imputation strategies
Per-protocol analysis	Does adherence materially affect the estimate?	Protocol deviations and contamination	Supports causal interpretation of the intervention
Severity-stratified subgroup analyses	Is benefit consistent across dysphagia severity?	Effect heterogeneity	Demonstrates equity and generalizability across severity strata
Ordinal outcome (poor/intermediate/good)	Does treatment shift patients across ordered health states?	Information loss from binary endpoint	Confirms direction and gradation of recovery

### Comparator endpoint: utility-weighted modified Rankin scale

To benchmark the sensitivity of the proposed dysphagia-focused PRO endpoint against a commonly used global stroke endpoint, we considered the utility-weighted modified Rankin Scale (UW-mRS) at 90 days, which combines the conventional mRS (0–6) with health utility weights to represent outcomes ranging from no disability to death (22, 64–66). On the UW-mRS, mRS categories are assigned utility weights and rescaled to a 0–10 metric, with higher scores indicating better health (64–66). Because dysphagia can substantially affect nutrition, aspiration risk, and psychosocial well-being without changing a patient’s mRS category, UW-mRS may be insensitive to dysphagia-specific recovery (67). Accordingly, UW-mRS was evaluated as a comparator endpoint in simulation analyses.

### Monte Carlo simulation study

To compare the sensitivity of alternative outcome measures for detecting therapeutic benefit in post-stroke dysphagia, we conducted Monte Carlo simulations intended to reflect clinically realistic recovery patterns and heterogeneity observed in acute stroke populations (68). A total of 10,000 patients with post-stroke dysphagia were simulated and randomly assigned 1:1 to control or treatment groups (5,000 per arm). Baseline dysphagia severity was categorized into low, moderate, and high strata with proportions of 35, 40, and 25%, respectively, reflecting distributions reported in observational cohorts (53). Severity was assumed to be determined early after stroke using the prespecified NIHSS-derived dysphagia severity score.

For each simulated participant, 90-day outcomes were generated for (1) SWAL-QOL (0–100 scale), (2) EQ-5D index value, and (3) UW-mRS. SWAL-QOL and EQ-5D values were generated using severity-specific normal distributions (27, 69). Treatment effects were modeled as minimal in the low-severity group (to reflect ceiling effects), moderate in the moderate-severity group, and largest in the high-severity group, consistent with expected mechanisms of dysphagia-targeted therapies. For UW-mRS, baseline mRS categories typical of dysphagia trial populations were sampled and mapped to utilities using published weights (70); only a small proportion of treated participants were allowed to improve by one mRS category to reflect limited sensitivity of global disability measures to dysphagia-specific recovery.

**Figure 2:**
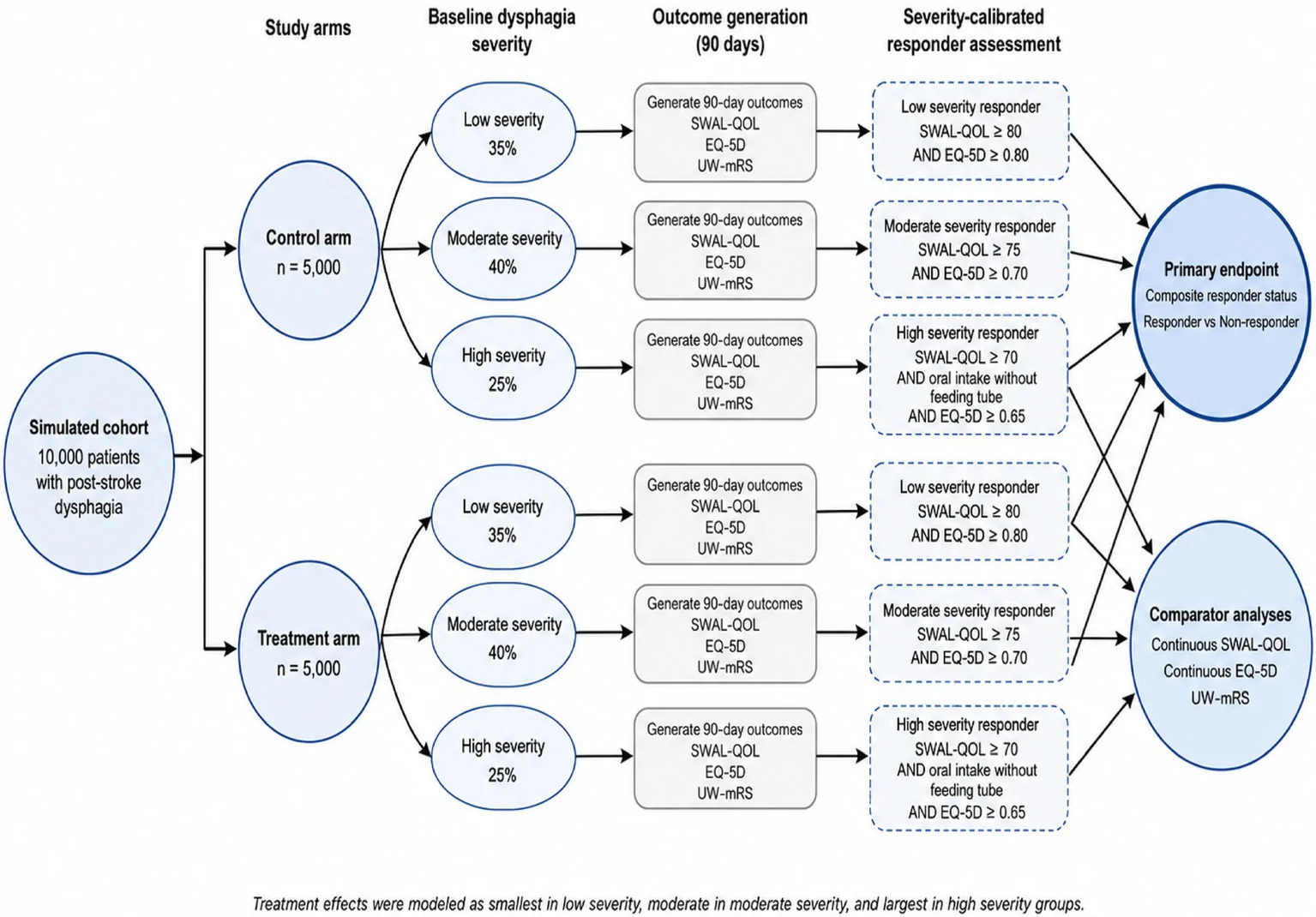
State transition diagram for the Monte Carlo simulation. A simulated cohort of 10,000 patients with post-stroke dysphagia was randomized 1:1 to control and treatment arms and stratified by baseline dysphagia severity (low, moderate, high). For each severity group, 90-day SWAL-QOL, EQ-5D, and UW-mRS outcomes were generated, and responder status was determined using severity-calibrated SWAL-QOL and EQ-5D thresholds. The primary endpoint was composite responder status, with continuous SWAL-QOL, continuous EQ-5D, and UW-mRS analyzed as comparators.

The primary responder endpoint was computed using the prespecified severity-calibrated status thresholds for SWAL-QOL and EQ-5D at 90 days (Table 3), with responder status requiring concordant success on both measures. Comparative performance of the combined responder endpoint was evaluated against continuous SWAL-QOL and UW-mRS analyses under the simulated distributions. Monte Carlo simulations were performed using Python (version 3.12) with the NumPy library for random number generation and data simulation, SciPy for statistical analyses, and Matplotlib for graphical visualization. Random number generation used a fixed seed to ensure reproducibility. Figure 2 shows state transition diagram for the Monte Carlo simulation.

## Results

### Concordance of SWAL-QOL and EQ-5D

In a simulated cohort reflecting typical post-stroke dysphagia recovery patterns, swallowing-specific quality of life (SWAL-QOL) and global health status (EQ-5D) showed only moderate concordance (Table 6). Under a liberal “either/or” definition, approximately three-quarters of patients would be classified as having a good outcome, but a substantial proportion of these responders would have clinically meaningful residual impairment in one domain. Requiring concordant success on both SWAL-QOL and EQ-5D reduced responder classification to approximately 40% in the base-case correlation scenario and reduced false-positive responder classification by approximately 30–60% across plausible correlations (rho = 0.3–0.7).

**Table 6:** Estimated concordance by assumed correlation.

Assumed correlation (*ρ*)	Both responders (%)	Either/Or responders (%)	False responders (%)	Relative reduction in false responders*
0.30 (low)	33%	82%	49%	60%
0.40	36%	79%	43%	54%
0.50 (base case)	40%	75%	35%	47%
0.60	44%	71%	27%	38%
0.70 (high)	48%	67%	19%	28%

*Relative reduction = (false responders under either/or)/(total responders under either/or).

### Monte Carlo simulation outcomes

Across simulated scenarios, the combined responder endpoint showed greater sensitivity to dysphagia-targeted therapeutic benefit than continuous PRO analyses or global disability measures. Overall, 42.7% of control participants met responder criteria compared with 66.2% of treated participants, corresponding to an absolute increase of 23.5 percentage points (Odds ratio (OR) 2.58, 95% Confidence interval (CI) 2.38–2.81; NNT approximately 4–5).

Treatment effects were largest in the moderate and high dysphagia severity strata, consistent with expected ceiling effects among mildly affected patients. In moderate dysphagia, responder rates increased from 16.4% (control) to 48.1% (treatment) (absolute difference 31.7%; OR 4.72, 95% CI 4.07–5.47). In severe dysphagia, responder rates increased from 0.7% to 24.5% (absolute difference 23.8%; OR 45.9, 95% CI 23.5–89.5). In mild dysphagia, responder rates increased from baseline by 6.7 percentage points (OR 1.32, 95% CI 1.16–1.52).

### Comparison with continuous SWAL-QOL and UW-mRS

Mean SWAL-QOL scores improved from 67.9 (control) to 76.2 (treatment), yielding a mean difference of 8.3 points. Although statistically significant in large, simulated samples, this average difference was near or below commonly cited thresholds for minimal clinically important improvement and was attenuated by ceiling effects among mildly affected participants.

The UW-mRS showed minimal sensitivity to dysphagia-specific treatment effects. Mean UW-mRS increased from 0.627 (control) to 0.644 (treatment), corresponding to a mean difference of 0.017. Power simulations suggested that detecting differences of this magnitude would require several thousand participants, making UW-mRS impractical as a primary endpoint for dysphagia-focused trials.

### Comparison with continuous outcomes

Compared with continuous mean-based analyses, the dichotomous severity-anchored responder endpoint provides improved interpretability and favorable power under realistic variance and missing-data conditions.

## Discussion

In this work, we propose and justify a patient-centered endpoint framework for clinical trials in post-stroke dysphagia that directly addresses longstanding methodological barriers in this field. Dysphagia recovery after stroke is heterogeneous, severity-dependent, and frequently accompanied by acute-phase instability that limits the reliability and feasibility of baseline patient-reported outcome assessment (41, 71). Traditional analytic approaches relying on mean or median score comparisons-or change from baseline-are therefore poorly suited to this context and risk underestimating clinically meaningful recovery, particularly among patients with severe dysphagia. A central contribution of this framework is the use of a status-based, dichotomous responder endpoint assessed at a standardized recovery milestone. Importantly, requiring concordant improvement in both swallowing-specific quality of life (SWAL-QOL) and global health status (EQ-5D) ensures that recovery reflects integrated functional improvement rather than isolated gains in a single domain. The AND composite prioritizes specificity by requiring improvement in both swallowing-related and global quality of life. However, it may underestimate dysphagia-specific benefit in patients whose SWAL-QOL improves meaningfully but whose EQ-5D remains limited by mobility impairment, dependence, or other stroke-related disabilities unrelated to swallowing. Therefore, the composite should be interpreted alongside its individual components, with SWAL-QOL serving as the more direct measure of dysphagia-specific recovery.

Simulation analyses demonstrated that liberal “either/or” definitions substantially overestimate benefit, whereas a dual-measure requirement reduces false-positive responder classification by approximately 30–60% across plausible correlations between domains. Anchoring responder definitions to NIHSS-derived dysphagia severity further strengthens interpretability and equity. Swallowing-relevant NIHSS subcomponents provide an objective, early, and widely available severity anchor that is independent of patient-reported feasibility and intervention timing (52, 53).

Persistent post-stroke dysphagia may require gastrostomy for long-term nutritional support, commonly through gastrostomy tube placement or radiologically inserted gastrostomy (RIG). A study found comparable times to goal feeding with both approaches, while RIG was associated with lower adjusted odds of reoperation, intensive care admission, and perioperative cerebrovascular events (72). These findings suggest that gastrostomy tube placement technique and patient comorbidities may influence procedural burden and global quality of life independently of swallowing recovery. Therefore, surgical or radiologic feeding-tube management should be documented and considered when interpreting SWAL-QOL, EQ-5D, and composite responder outcomes.

Monte Carlo simulation was used to compare the sensitivity and interpretability of alternative endpoint strategies under clinically realistic conditions for post-stroke dysphagia, providing a transparent method to isolate the impact of endpoint choice while holding the underlying treatment effect constant. The simulation incorporated real-world features of stroke populations, including heterogeneity in dysphagia severity, ceiling and floor effects, and differential recovery trajectories. Under these conditions, dysphagia-targeted therapeutic benefit was poorly captured by global disability measures such as the UW-mRS, which showed only minimal changes despite substantial swallowing recovery and required several thousand participants to detect a statistically significant effect. Continuous SWAL-QOL analyses detected differences only at very large sample sizes and underestimated recovery among patients with moderate and severe dysphagia due to ceiling effects in mildly affected patients. In contrast, an NIHSS-anchored combined SWAL-QOL and EQ-5D responder endpoint consistently demonstrated the greatest sensitivity to therapeutic benefit, with large and clinically interpretable increases in responder rates across severity strata that persisted under conservative assumptions regarding treatment effect and variance, supporting its selection as the primary endpoint for post-stroke dysphagia trials. From a statistical perspective, responder-based analyses demonstrated superior efficiency under realistic assumptions. While continuous endpoints may appear efficient under optimistic variance assumptions, such conditions are rarely met in acute stroke dysphagia, where variance inflation, bimodal distributions, and missing baselines are common (73). In contrast, the NIHSS-anchored responder endpoint maintained stable sample size requirements and achieved adequate power with fewer participants under real-world conditions. These advantages persisted in both individually randomized and stepped-wedge designs, supporting applicability across explanatory and pragmatic trial settings.

### Limitations and future directions

Several limitations should be acknowledged. First, the proposed responder thresholds and NIHSS-based dysphagia severity calibration are derived from existing literature, expert consensus, and simulation-based evaluation rather than prospective validation within a single large cohort. Although this approach is consistent with accepted outcomes-development methodology, empirical validation of these thresholds against long-term clinical outcomes, patient priorities, and caregiver-reported burden will further strengthen their generalizability. Second, while the combined SWAL-QOL and EQ-5D responder endpoint enhances specificity and interpretability by requiring concordant improvement across disease-specific and global domains, this stringency may underestimate benefit in patients who experience meaningful improvement in a single domain. For this reason, analyses of individual PRO components and continuous outcomes remain important as secondary and supportive endpoints, particularly for mechanistic interpretation and hypothesis generation. Third, the dysphagia severity score is anchored to selected NIHSS subcomponents that are strongly associated with swallowing dysfunction; however, NIHSS was not originally designed as a dysphagia instrument (49, 52). Although this pragmatic anchoring improves feasibility and equity in acute stroke trials, future work should explore integration with objective swallowing assessments, imaging biomarkers, or digital phenotyping approaches to refine severity stratification further. Finally, the proposed framework was developed primarily with randomized clinical trials in mind and assumes assessment at a standardized recovery milestone (90 days). Its performance in alternative contexts-such as very early recovery windows, chronic stroke populations, or health-system-level implementation studies-warrants further study (74). Accordingly, the proposed thresholds should be considered hypothesis-generating and require prospective validation and sensitivity testing before being used as definitive responder criteria.

Despite these limitations, the framework provides a transparent, patient-centered, and methodologically robust starting point for evaluating new therapeutic interventions in post-stroke dysphagia. Future directions include prospective validation of responder definitions, harmonization across international stroke registries, and incorporation into adaptive and pragmatic trial designs aimed at accelerating therapeutic development in this high-burden condition. Adoption of this framework has the potential to improve trial efficiency, enhance interpretability, and accelerate development of effective interventions for a complication that remains a major unmet need in stroke care. Future observational studies and embedded trial analyses should examine how severity-calibrated responder status relates to downstream outcomes such as aspiration pneumonia, institutionalization, and health-care utilization (75).

## Conclusion

A severity-calibrated, status-based PRO responder endpoint assessed at 90 days and requiring concordant improvement in SWAL-QOL and EQ-5D offers a patient-centered, interpretable, and statistically efficient framework for evaluating interventions in post-stroke dysphagia. Anchoring responder thresholds to NIHSS-derived dysphagia severity supports equitable interpretation across the heterogeneous severity spectrum typical of acute stroke populations.

## Data Availability

The original contributions presented in the study are included in the article/Supplementary material, further inquiries can be directed to the corresponding author.
