# Supplementary material for: A severity-anchored, patient-reported outcome framework for clinical trials in post-stroke dysphagia
Source: Front Neurol. 2026 Aug 24;17:1826727. doi: 10.3389/fneur.2026.1826727 (PMC13547051; doi:10.3389/fneur.2026.1826727)
Supplement: Supplementary file 1 [file Table_1.docx]

# Figure legends and supplementary material

Supplemental Figure 1.

Proportion of patients achieving swallowing-specific (SWAL-QOL), global health-related (EQ-5D), and combined responder outcomes at 90 days post-stroke. While modest differences are observed for individual patient-reported outcomes, the combined responder endpoint-requiring concordant improvement in both domains-demonstrates a substantially larger separation between treatment groups, reflecting integrated and clinically meaningful recovery.
